# A KSA system for competency-based assessment of clinicians’ professional development in China and quality gap analysis

**DOI:** 10.1080/10872981.2022.2037401

**Published:** 2022-02-09

**Authors:** Xiaoqing Huang, Zihua Li, Jiali Wang, Endong Cao, Guiying Zhuang, Fei Xiao, Caihua Zheng, Xiaowen Zhang, Man Chen, Liqing Gao, Pi Guo, Peiwei Lin, Shaoyan Zheng, Gang Xin

**Affiliations:** a Department of Clinical Medicine, Shantou University Medical College, Guangdong, China; b Department of Public Health and Preventive Medicine, Shantou University Medical College, Guangdong, China; c Department of Network Information Center, Shantou University Medical College, Guangdong, China; d Department of Teaching Affairs Office, Shantou University Medical College, Guangdong, China; e Department of Microbiology and Immunology, Shantou University Medical College, Guangdong, China

**Keywords:** Core competency, medical graduate, educational assessment, service quality model, quality gap analysis

## Abstract

**Background:**

We aim to create a holistic competency-based assessment system to measure competency evolution over time – one of the first such systems in China.

**Method:**

Two rounds of self-reported surveys were fielded among the graduates from the Shantou University Medical College: June through December 2017, and May through August 2018. Responses from three cohorts of graduates specializing in clinical medicine – new graduates, resident physicians, and senior physicians – were analyzed. Gaps between respondents’ expected and existing levels of competencies were examined using a modified service quality model, SERVQUAL

**Results:**

A total of 605 questionnaires were collected in 2017 for the construction of competency indicators and a 5-level proficiency rating scale, and 407 in 2018, for confirmatory factor and competency gap analysis. Reliability coefficients of all competency indicators (36) were greater than 0.9. Three competency domains were identified through exploratory factor analysis: knowledge (K), skills (S), and attitude (A). The confirmatory factor analysis confirmed the fit of the scale (CMIN/DF < 4; CFI > 0.9; IFI > 0.9; RMSEA ≤ 0.08). Within the cohorts of resident and senior physicians, the largest competency gap was seen in the domain of knowledge (K): −1.84 and −1.41, respectively. Among new graduates, the largest gap was found in the domain of skills (S) (−1.92), with the gap in knowledge (−1.91) trailing closely behind.

**Conclusions:**

A competency-based assessment system is proposed to evaluate clinician’s competency development in three domains: knowledge (K), skills (S), and attitude (A). The system consists of 36 competency indicators, a rating scale of 5 proficiency levels, and a gap analysis to measure competency evolution through 3 key milestones in clinician’s professional career: new graduate, resident physician, and senior physician. The competency gaps identified can provide evidence-based guide to clinicians’ own continuous development as well as future medical curriculum improvements.

## Introduction

Epstein and Hundert [1,2] defined systems-based competencies for health professionals as follows: ‘the habitual and judicious use of communication, knowledge, technical skills, clinical reasoning, emotions, values, and reflection in daily practice for the benefit of the individual and the community being served.’ It was further advocated in the report entitled ‘Health Professionals for a New Century: Transforming Education to Strengthen Health Systems in an Interdependent World’ and published by the Lancet Commissions in 2010 that ‘a 3^rd^ generation [of medical education] is now needed that should be systems based to improve the performance of health systems by adapting core professional competencies to specific contexts while drawing on global knowledge.’[3]

The earliest literature on physician core competencies can be traced as far back as to the 1970s. Government agencies and organizations worldwide have since been continuously updating these competencies. In 1998, the Accreditation Council for Graduate Medical Education (ACGME) in the U.S. defined core competencies in 6 areas for health practitioners[4]. In 2005, the Royal College of Physicians and Surgeons of Canada published the CanMEDS 2005 Physician Competency Framework as an update to the previous version (published in 1996 and entitled ‘Skills for the New Millennium’), outlining 7 physician roles[5]. In 2013, the General Medical Council in UK issued a guidance document entitled ‘Good Medical Practice’ to delineate the duties of doctors[6].

In order to transform medical education for the twenty-first century, it is essential that educational institutes (medical colleges and schools, teaching hospitals, etc.) strengthen their faculty teams and promote curriculum reforms to elevate a broad range of capabilities of the medical personnel [3,7]. Graduate surveys that collect feedback from the recipients of medical education have been relied upon as one of the effective tools to gauge the teaching quality at medical institutes, and can provide valuable input to help direct plans to improve medical curricula [8,9].

Since 1998, China has implemented the largest reform of medical education in the world by incorporating professional training into college education. This has significantly boosted the enrollment of health-care professionals at medical institutes[7]. In 2015, standardized resident training was also introduced in China[10]. There are three main tracks of formal medical education in China, which aspiring high-school graduates can pursue: the 5-year, the 5 + 3, and the 8-year programs. For the 5-year track, high-school graduates enroll themselves to the undergraduate medical program and will receive a bachelor’s degree at the end of their 5 years of study (‘new graduate’). These new graduates are eligible for standardized resident training which will last another 3 years. For the 5 + 3 track, after students complete their initial 5-year training (equivalent to that of the 5-year track), they attend a 3-year standardized resident training and will be awarded a master’s degree together with a standardized resident training certificate (‘resident physicians’) when completing the program. For the 8-year track, high-school graduates attend a broader training program that spans basic and clinical medicine as well as liberal arts, and will receive a degree of MD (Doctor of Medicine/Medical Doctor) at the end of their 8 years of study. Like ‘new graduates’, MDs can take up additional standardized resident training which will last 2 to 3 years. The bachelor’s degree prepares new graduates for a career in clinical medicine, if they so choose, or related professions. The goal of the 5 + 3 training program is to cultivate a pipeline of clinical physicians, while the 8-year program aims to incubate medical talents with more versatility.

From 2012 to 2014, Dr. Baozhi Sun, former Vice President of the China Medical University, joined efforts with a team of scholars to conduct a large-scale and cross-sectional survey among clinicians in 31 provinces and cities across the country. They constructed the ‘Chinese Doctors’ Common Competency Model’ which consists of 76 indicators and covers 3 key dimensions of competency knowledge, skills, and attitude (KSA). The model encompasses the following aspects of medicine: clinical skills and patient care, disease prevention and health promotion, information and management, medical knowledge and life-long learning, interpersonal communication, teamwork and scientific research, core value, and professionalism. However, the model developed by Sun et al. mainly targets senior physicians with more extensive clinical experience as practitioners.

Nevertheless, holistic systems to assess medical graduates’ professional development as they progress through different phases of their career remain few and far between in China. What is also lacking is a keen appreciation of professional development as a continuous and dynamic process, as well as investigations to assess this process that are reproducible. Therefore, the objective of the current study is to create a holistic competency-based assessment system comprising 3 components: competency indicators suitable for clinicians in different phases of their career, a rating scale aligned with the progression of skill acquisition, and an analytical tool to measure competency evolution over time – one of the first such systems in China.

## Method

A competency-based KSA assessment system was designed by drawing from the conceptual framework of Norcini who espoused that an effective assessment system should include three segments: competency (defined by indicators), level of assessment (degree of mastery), and assessment of progression (skill acquisition through stages) [2,11,12]. The Dreyfus model that any skill acquisition spans 5 stages – novice, advanced beginner, competent, proficient, and expert [13] – was also consulted to create a more nuanced scheme for assessing the mastery level of competency. This study was approved by the Ethics Committee of the Shantou University of Medical College (SUMC).

### KSA-based competency indicators and a rating scale

**Figure 1.**
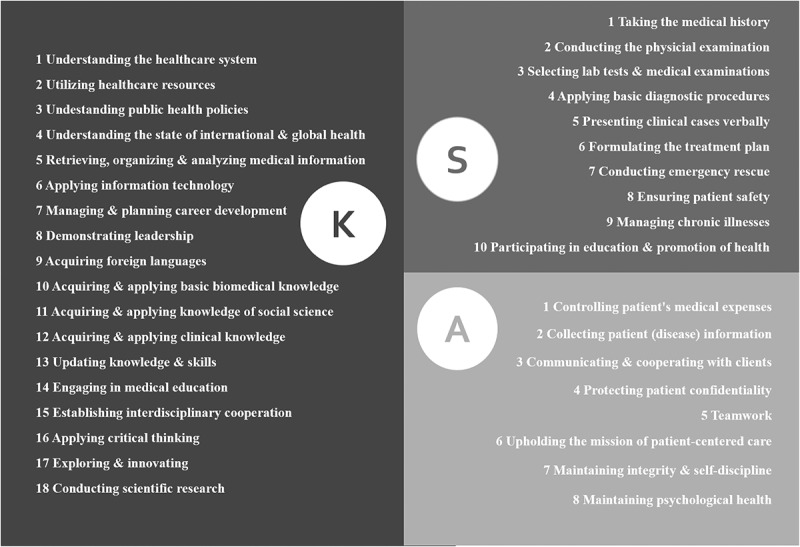
KSA Model of Core Competencies (36 indicators).

Thirty-six indicators (Figure 1) were derived by combining and simplifying closely related indicators from the model created by Sun et al [2]. so the scale can be applicable for surveying a more diverse group of clinicians – that is, new graduates, resident physicians, and senior physicians – who were selected to represent 3 key milestones in a clinician’s professional career. A more succinct scale also rendered the survey less cumbersome to administer, more enticing for respondents to complete the survey, thereby allowing the collection of more meaningful data.

The questionnaire developed based on this scale includes two sections: basic information, and self-assessment of competencies. The self-assessment is based on the 5-point Likert scale defined as follows: 0 represents ‘do not know’; ‘1’ represents ‘beginner’ (having acquired cognitive understanding of the relevant basics); ‘2’ represents ‘application’ (being able to practice or simulate under the guidance of others; ‘3’ represents ‘competent’ (being able to practice independently in the real world according to standards; ‘4’ represents ‘proficient’ (being able to practice independently and deliver top-quality outcome); and ‘5’ represents ‘expert’ (being able to serve as an example for peers and in an advisory capacity as well as participate in developments of standards). Respondents were asked to rate both their existing and expected levels of competencies.

The anonymous questionnaire was made available on the graduate survey platform (http://bysczdc.med.stu.edu.cn/) at the SUMC from June through December 2017. All participants had earned a degree in clinical medicine from the SUMC or partnering hospitals. Responses from those enrolled in 2012 (‘new graduates’), 2008/2009 (‘resident physicians’), or 2005/2006 (‘senior physicians’) were analyzed for the construction of competency indicators. Respondents were informed that their answers would be kept strictly confidential and that they could withdraw from the survey at will. All participants completed and submitted the questionnaires electronically or on paper.

Questionnaires collected were excluded from analysis if they met any of the following criteria: from graduates who earned their degrees outside the 3 time points specified; from respondents who no longer worked in the field of clinical medicine; from those who populated the answers mechanically (e.g., filled each question with identical answers); from respondents who submitted multiple questionnaires using the same Internet Protocol (IP) address (in this case, the last questionnaire submitted would be treated as valid input, with the rest, discarded).

SPSS Statistics 21.0 for Windows (IBM Corp., Armonk, New York) was used to analyze reliability and validity. The competency level of ‘0’ was equated as ‘missing data’ and substituted with the mean score (‘mean imputation’)[14]. The Cronbach’s alpha value was used to evaluate the internal consistency. The Kaiser–Meyer–Olkin (KMO) measure greater than 0.9 and the significance level of Bartlett’s test of sphericity less than 0.05 would indicate that the data were suitable for exploratory factor analysis (EFA)[15]. Factors with eigenvalues greater than 1 and factor loading greater than 0.45 would be extracted after orthogonal rotation with Kaiser normalization. If there were multiple-factor loadings greater than 0.45, the factor with the highest loading would be selected[16].

For the confirmatory factor analysis, a separate random survey (using the same questionnaire) was fielded from May through August in 2018 among graduates who enrolled in the clinical medicine department at the SUMC in 2013 (‘new graduates’), 2010 (‘resident physicians’), or 2007 (‘senior physicians’). Confirmatory factor analysis using the software Amos 21.0 for Windows (IBM Corp., Armonk, New York) was carried out to test the fit of the scale. The reasonable fit of the scale would be determined based on the following: chi-square to the degree of freedom ratio (CMIN/DF) < 4; comparison fit index (CFI) > 0.9; incremental fit index (IFI) > 0.9; and root mean square error of approximation (RMSEA) ≤ 0.08[17].

### Gap analysis of competencies and perceived quality of medical education by graduates

A revised service quality model, SERVQUAL – which was originally designed for commercial applications to business services [17] – was employed for the competency gap analysis based on the same survey responses collected in 2018. The quality of medical education (as measured by the gap between the existing competency level and the expected level) for the i^th^ indicator is represented by $Q_{i}={\overset{ˉ}{P}}_{i}-{\overset{ˉ}{E}}_{i}$, where *P_i_* indicates the perceived existing level of competency for the i^th^ indicator, and *E_i_*, the expected level of competency[18]. The quality of medical education for each of the KSA domain is $Q=\frac{1}{m}$$\sum_{i=1}^{m}\left({\overset{ˉ}{P}}_{i}-{\overset{ˉ}{E}}_{i}\right)$, where *m* represents the number of indicators in each domain. When *m* = 36, Q indicates the overall quality of medical education. The Kruskal–Wallis test was used to analyze the differences in perceived quality among the three groups of respondents.

## Results

### The KSA-based competency indicators

**Reliability and validity**. There were 226, 193, and 186 questionnaires collected from new graduates, resident physicians, and senior physicians, respectively, which were included according the established criteria (Table 1). The Cronbach’s alpha values (reliability coefficients) for each item in the questionnaire and the questionnaire as a whole were both greater than 0.9. Therefore, all 36 core competency indicators were retained. The KMO values associated with the 3 groups of respondents were 0.967, 0.964, and 0.943, respectively. The *p* values of the Bartlett’s sphericity test were less than 0.001. The indicators were thus suitable for factor analysis.

**Table 1.** A Summary of Questionnaire Responses

Analysis Supported		Male (*n*)	Female (*n*)	Total (*n*)
Construction of Indicators	New Graduates	113 (50.0%)	113 (50.0%)	226
	Resident Physicians	114 (59.1%)	79 (40.9%)	193
	Senior Physicians	109 (58.6%)	77 (41.4%)	186
Competency Gap Analysis	New Graduates	96 (60.4%)	63 (39.6%)	159
	Resident Physicians	86 (68.3%)	40 (31.7%)	126
	Senior Physicians	75 (61.5%)	47 (38.5%)	122

Based on the exploratory factor analysis, 3, 3, and 5 factors were extracted from the groups of new graduates, resident physicians, and senior physicians, respectively (Table 2). Three out of the five factors extracted from senior physicians shared the same constructs and were combined into one single factor (i.e., ‘knowledge’). The Cronbach’s alpha values for all factors associated with each group were greater than 0.9, indicating high internal consistency. The factors extracted were analyzed further, and three domains emerged with which the competency indicators measured were aligned: knowledge (K), skills (S), and attitude (A).

**Table 2.** Rotated Component Matrix of Exploratory Factor Analysis^b,c^

Indicator	New Graduate			Resident Physician			Senior Physician					Corresponding KSA Indicator^d^
	K^a^	A^a^	S^a^	K	S	A	S	K	A	K	K	
1	0.15	0.53	0.61	0.16	0.79	0.38	0.77	0.16	0.31	0.07	<0.001	S1
2	0.21	0.43	0.75	0.21	0.80	0.33	0.80	0.20	0.18	0.15	0.10	S2
3	0.43	0.33	0.70	0.28	0.72	0.32	0.74	0.22	0.32	0.13	0.13	S3
4	0.42	0.39	0.68	0.35	0.74	0.36	0.75	0.24	0.36	0.12	0.16	S4
5	0.46	0.34	0.68	0.29	0.74	0.39	0.81	0.20	0.28	0.19	0.05	S5
6	0.56	0.26	0.65	0.32	0.74	0.35	0.86	0.08	0.19	0.20	0.15	S6
7	0.64	0.10	0.47	0.41	0.67	0.25	0.64	0.18	0.12	0.29	0.22	S7
8	0.50	0.38	0.53	0.33	0.70	0.39	0.69	0.09	0.35	0.21	0.20	S8
9	0.64	0.17	0.53	0.48	0.64	0.22	0.52	0.09	0.15	0.56	0.27	S9
10	0.57	0.40	0.44	0.53	0.60	0.25	0.39	0.20	0.23	0.65	0.13	S10
11	0.74	0.20	0.41	0.64	0.58	0.15	0.25	0.33	0.11	0.75	0.14	K1
12	0.73	0.23	0.43	0.66	0.57	0.14	0.29	0.34	0.19	0.75	0.08	K2
13	0.79	0.12	0.31	0.77	0.38	0.13	0.16	0.44	0.19	0.71	0.18	K3
14	0.84	0.20	0.22	0.73	0.38	0.01	0.08	0.51	0.09	0.62	0.22	K4
15	0.76	0.29	0.37	0.66	0.31	0.33	0.26	0.81	0.18	0.23	0.03	K5
16	0.73	0.27	0.30	0.70	0.26	0.38	0.31	0.75	0.23	0.19	0.02	K6
17	0.79	0.17	0.18	0.56	0.40	0.44	0.22	0.34	0.48	0.37	0.15	A1
18	0.78	0.28	0.22	0.69	0.37	0.42	0.08	0.56	0.29	0.49	0.27	K7
19	0.77	0.34	0.19	0.77	0.23	0.37	0.11	0.62	0.20	0.34	0.22	K8
20	0.58	0.39	0.28	0.74	0.21	0.30	0.19	0.72	0.02	0.09	0.26	K9
21	0.70	0.33	0.37	0.80	0.19	0.30	0.10	0.77	0.12	0.18	0.25	K10
22	0.74	0.32	0.26	0.79	0.32	0.32	0.05	0.69	0.26	0.35	0.15	K11
23	0.56	0.50	0.46	0.54	0.40	0.55	0.40	0.44	0.49	0.22	0.18	K12
24	0.65	0.46	0.40	0.61	0.31	0.59	0.33	0.52	0.34	0.25	0.30	K13
25	0.77	0.34	0.22	0.71	0.21	0.39	0.24	0.62	0.20	0.29	0.28	K14
26	0.35	0.64	0.50	0.32	0.48	0.66	0.49	0.24	0.56	0.15	0.12	A2
27	0.46	0.65	0.40	0.35	0.44	0.69	0.46	0.22	0.64	0.15	0.12	A3
28	0.21	0.79	0.32	0.26	0.44	0.72	0.45	0.06	0.73	0.11	0.06	A4
29	0.50	0.64	0.22	0.44	0.35	0.62	0.27	0.12	0.59	0.16	0.48	A5
30	0.70	0.46	0.19	0.57	0.27	0.51	0.15	0.24	0.25	0.26	0.73	K15
31	0.63	0.57	0.23	0.67	0.29	0.45	0.25	0.28	0.35	0.20	0.57	K16
32	0.70	0.52	0.22	0.70	0.32	0.41	0.20	0.40	0.24	0.24	0.70	K17
33	0.76	0.31	0.26	0.78	0.25	0.33	0.15	0.51	0.02	0.08	0.65	K18
34	0.42	0.73	0.28	0.46	0.40	0.64	0.30	0.17	0.73	0.13	0.27	A6
35	0.16	0.84	0.20	0.22	0.30	0.74	0.22	0.16	0.75	0.07	0.07	A7
36	0.21	0.82	0.22	0.32	0.31	0.68	0.24	0.20	0.70	0.24	0.14	A8

^a^K: Knowledge; S: Skills; A: Attitude;.

^b^Extraction method: Principal component analysis.

^c^Rotation method: Varimax with Kaiser normalization (Rotation converged in 8 iterations).

^d^These indicators are detailed in Figure 1.

**Confirmatory factor analysis**. There were 159, 126, and 122 questionnaires collected from the 3 cohorts of respondents, respectively, that were included according the established criteria (Table 1). The reasonable fit of the scale was confirmed based on the following: CMIN/DF = 3.596; CFI = 0.905; IFI = 0.905; RMSEA = 0.080.

### Gap analysis of competencies and perceived quality of medical education by graduates

As shown in Table 3, the Q values represent the gaps between the existing and expected levels of competency as perceived by the 3 groups of participating graduates. Q values are negative for all 36 core competency indicators. Based on the total Q values, the largest overall competency gap is found among new graduates (−1.81), followed by resident physicians (−1.70) and senior physicians (−1.29) in that order. Within individual cohorts, the largest gap among resident physicians (−1.84) and senior physicians (−1.41) is seen in the domain of knowledge (K). Among new graduates, the largest gap (−1.92) is associated with the domain of skills (S), with the gap (−1.91) in knowledge trailing closely behind. For the domain of skills (S), both resident and senior physicians perceive their existing competency levels to be merely ‘applicable’ (2.46 & 2.77, respectively), which contrasts starkly with their expected levels of ‘proficient’ (4.13 & 4.02, respectively). New graduates, on the other hand, view their existing level as ‘applicable’ (2.00), while expecting their competency to reach the level of ‘competent’ (3.92).

**Table 3.** Competency Gap Analysis Based on the Modified SERVQUAL Model

Indicator^b^	New Graduate					Resident Physician					Senior Physician				
	P^a^	E^a^	Q^a^	Paired Sample Test		P	E	Q	Paired Sample Test		P	E	Q	Paired Sample Test	
				*t*	*p*				*t*	*p*				*t*	*p*
K1	1.72	3.47	−1.75	−21.29	<0.001	2.01	3.75	−1.74	−18.47	<0.001	2.39	3.65	−1.26	−13.52	<0.001
K2	1.72	3.54	−1.82	−21.66	<0.001	1.98	3.80	−1.83	−19.59	<0.001	2.35	3.70	−1.35	−14.75	<0.001
K3	1.55	3.49	−1.94	−24.14	<0.001	1.97	3.75	−1.78	−19.11	<0.001	2.30	3.66	−1.37	−14.24	<0.001
K4	1.53	3.38	−1.86	−22.39	<0.001	1.85	3.63	−1.78	−18.93	<0.001	2.12	3.56	−1.44	−14.86	<0.001
K5	1.68	3.72	−2.04	−25.11	<0.001	2.05	3.93	−1.89	−19.97	<0.001	2.14	3.66	−1.52	−14.84	<0.001
K6	1.71	3.72	−2.00	−23.78	<0.001	2.02	3.89	−1.88	−19.84	<0.001	2.15	3.60	−1.45	−14.93	<0.001
K7	1.69	3.59	−1.91	−23.64	<0.001	1.91	3.86	−1.95	−20.36	<0.001	2.09	3.63	−1.53	−15.51	<0.001
K8	1.72	3.56	−1.84	−22.04	<0.001	1.93	3.81	−1.88	−19.63	<0.001	1.95	3.50	−1.56	−15.32	<0.001
K9	1.86	3.83	−1.97	−22.20	<0.001	1.96	3.88	−1.92	−20.87	<0.001	1.97	3.61	−1.64	−15.34	<0.001
K10	1.75	3.64	−1.89	−24.17	<0.001	1.92	3.80	−1.88	−19.50	<0.001	2.01	3.45	−1.44	−14.80	<0.001
K11	1.68	3.58	−1.90	−23.47	<0.001	1.98	3.75	−1.76	−19.56	<0.001	2.15	3.45	−1.30	−14.03	<0.001
K12	1.85	3.89	−2.04	−23.84	<0.001	2.27	4.05	−1.78	−19.83	<0.001	2.52	3.79	−1.27	−14.71	<0.001
K13	1.81	3.82	−2.01	−21.98	<0.001	2.21	3.98	−1.77	−19.58	<0.001	2.39	3.66	−1.27	−14.33	<0.001
K14	1.61	3.46	−1.85	−20.75	<0.001	1.99	3.78	−1.79	−18.94	<0.001	2.12	3.53	−1.41	−14.47	<0.001
K15	1.65	3.39	−1.74	−20.28	<0.001	1.89	3.80	−1.91	−20.46	<0.001	2.05	3.53	−1.49	−15.20	<0.001
K16	1.79	3.67	−1.88	−23.84	<0.001	2.15	3.87	−1.71	−19.10	<0.001	2.19	3.50	−1.31	−14.16	<0.001
K17	1.69	3.63	−1.94	−24.32	<0.001	2.00	3.86	−1.86	−19.98	<0.001	2.09	3.46	−1.38	−14.96	<0.001
K18	1.56	3.58	−2.02	−23.63	<0.001	1.88	3.80	−1.92	−20.75	<0.001	1.92	3.38	−1.47	−14.52	<0.001
S1	2.49	4.11	−1.62	−22.11	<0.001	2.80	4.22	−1.43	−16.41	<0.001	2.97	4.06	−1.09	−13.81	<0.001
S2	2.26	4.11	−1.85	−23.90	<0.001	2.66	4.22	−1.56	−17.67	<0.001	2.85	4.06	−1.20	−14.50	<0.001
S3	2.03	3.97	−1.94	−23.44	<0.001	2.58	4.14	−1.56	−16.86	<0.001	2.83	4.06	−1.23	−16.15	<0.001
S4	2.04	3.98	−1.94	−23.31	<0.001	2.58	4.18	−1.60	−18.07	<0.001	2.87	4.09	−1.22	−15.06	<0.001
S5	1.96	3.94	−1.98	−24.45	<0.001	2.50	4.18	−1.68	−18.66	<0.001	2.88	4.08	−1.20	−14.23	<0.001
S6	1.81	3.92	−2.11	−23.61	<0.001	2.42	4.18	−1.77	−19.64	<0.001	2.78	4.07	−1.29	−15.72	<0.001
S7	1.65	3.77	−2.12	−24.84	<0.001	2.15	4.09	−1.93	−19.85	<0.001	2.63	3.99	−1.36	−16.56	<0.001
S8	1.95	3.83	−1.88	−23.24	<0.001	2.39	4.10	−1.71	−18.64	<0.001	2.79	4.06	−1.27	−15.75	<0.001
S9	1.81	3.77	−1.96	−23.72	<0.001	2.21	4.02	−1.80	−19.50	<0.001	2.58	3.90	−1.32	−15.99	<0.001
S10	1.97	3.74	−1.77	−21.57	<0.001	2.25	3.99	−1.74	−18.38	<0.001	2.56	3.84	−1.29	−14.84	<0.001
A1	1.67	3.17	−1.51	−17.14	<0.001	1.97	3.49	−1.53	−15.04	<0.001	2.48	3.72	−1.25	−15.40	<0.001
A2	2.36	3.92	−1.56	−19.97	<0.001	2.43	4.01	−1.58	−16.38	<0.001	2.72	3.82	−1.10	−13.56	<0.001
A3	2.23	3.86	−1.62	−20.25	<0.001	2.45	4.06	−1.61	−17.34	<0.001	2.68	3.92	−1.24	−15.06	<0.001
A4	2.59	3.86	−1.27	−16.29	<0.001	2.73	4.02	−1.28	−13.97	<0.001	3.02	3.80	−0.79	−10.40	<0.001
A5	2.21	3.86	−1.65	−20.50	<0.001	2.41	3.98	−1.58	−15.46	<0.001	2.53	3.83	−1.30	−14.68	<0.001
A6	2.23	3.77	−1.54	−19.24	<0.001	2.51	3.95	−1.44	−13.66	<0.001	2.80	3.84	−1.03	−11.86	<0.001
A7	2.73	3.87	−1.14	−12.99	<0.001	2.96	4.04	−1.08	−11.34	<0.001	3.17	3.79	−0.61	−9.00	<0.001
A8	2.63	3.86	−1.23	−15.38	<0.001	2.70	4.02	−1.32	−13.07	<0.001	2.58	3.75	−1.17	−12.44	<0.001
K	1.70	3.61	−1.91	-	-	2.00	3.83	−1.84	-	-	2.16	3.57	−1.41	-	-
S	2.00	3.92	−1.92	-	-	2.46	4.13	−1.68	-	-	2.77	4.02	−1.25	-	
A	2.33	3.77	−1.44	-	-	2.52	3.95	−1.43	-	-	2.75	3.81	−1.06	-	
Overall	1.92	3.73	−1.81	-	-	2.24	3.94	−1.70	-	-	2.46	3.75	−1.29	-	-

^a^P: Perceived current level of competency; E: Perceived expected level of competency; Q = P – E (Gap between the perceived current and perceived expected competency levels).

^b^These indicators are detailed in Figure 1.

## Discussion

Unlike previous research that focused on such parameters as tangibility, reliability, responsiveness, assurance of services as well as empathy of the faculty and staff [19,20], our study aimed to evaluate the evolution of medical graduates’ core competencies in 3 domains: knowledge (K), skills (S), and attitude (A). We designed a competency-based assessment system that is holistic and implementable to examine how clinicians’ competencies have evolved from when they were new medical graduates, through residency, to becoming seasoned practicing physicians. The gap analysis, another component of our system, yielded uniquely valuable insights about the quality of medical education as perceived by the participating graduates.

In Table 3, the negative Q values for all 36 competency indicators among the 3 cohorts of graduates suggest a higher expected level of competency than participants’ perceived existing level. Based on the total Q values, the largest overall competency gap is seen among new graduates, followed by residents and senior physicians, in that order. In terms of domains, distinct gaps are found in domains of skills (S) and knowledge (K) in all 3 cohorts. Hence, there appear cohort-specific and domain-specific contributors to these gaps, and targeted remedial measures will be needed to bridge the gaps. For example, at the indicator level, the biggest gap among new graduates is associated with ‘conducting emergency rescue’ followed by ‘formulating the treatment plan’ – both indicators fall within the domain of skills (S). To bridge the gap, additional class hours – as part of the clinical skill training series at the SUMC – can be devoted to scenario-based simulation training. At the domain level, the biggest gap is found in the domain of skills (S) among new graduates. As required by laws and clinical practice standards in China, all medical activities shall be conducted under the supervision of senior physicians to ensure the safety of patients and the learning environment of medical students. New graduates can thus only reach the level where they can ‘apply’ the knowledge learned, but cannot reach the ‘competent’ level where they follow standard guidelines and practice independently. The ‘competent’ level of competency is now a requirement for standardized resident trainings in China. Therefore, there is a more urgent need to ramp up new graduates’ clinical skills, so they can be better prepared as they transition to the residency phase where more emphasis is placed on clinical practices. Methods such as simulation technique, standardized patient, and enhanced clinical exposure can all help elevate new graduates’ clinical skills [21,22].

Different levels of expectation were also found between new graduates and residents/senior physicians. While new graduates hoped to reach the level of ‘competent’ (competency level = 3) for the great majority of indicators when they graduated, resident & senior physicians aspired to become ‘proficient’ (competency level = 4) for more indicators. This difference is not a total surprise, given the different professional development phases that these graduates find themselves in. However, upon a closer examination, the expectation of ‘being proficient’ appeared predominantly associated with the domain of skills (‘S’) among residents (9 out of 10 skill-related indicators) and senior physicians (8 out of 10 skill-related indicators), and, to a lesser degree, among new graduates (2 out of 10 skill-related indicators). Interestingly, this strong correlation was not seen with the domains of attitude (‘A’) or knowledge (‘K’). In other words, the study participants did not demonstrate a similar degree of expectation for attitude- and knowledge-related competencies. This gravitation toward skill-defined competencies may reflect a paradigmatic orientation among medical graduates from the SUMC as a whole – which places higher emphasis on ‘skill acquisition’ than development of competencies in softer areas such as attitude and knowledge. This finding highlights the need to drive home not only the ultimate goal of nurturing well-rounded health-care professionals but also the importance of operationalizing this aim, so professional expectations can be raised accordingly and training courses/programs fit to deliver on this goal will be created and propagated. Fulfilling this objective also underscores the construction of a multi-component assessment system as proposed by this study to measure the multiple dimensions of medical competency.

### Implications

The competency-based assessment system that we propose can be completed not only by ‘receivers’ of medical education/training (e.g., medical graduates, as in the current study), but also ‘administers’ (e.g., instructors, supervisors) and ‘beneficiaries’ (e.g., patients) of this education/training (although some modification of the indicators may be needed for the survey to be more meaningful to the latter group of stakeholders). This broad set of potential applications can facilitate the creation of a 360-degree survey of clinician’s core competencies, which echoes the systems-oriented characterization of the competencies that physicians need to demonstrate in order to serve the health-care needs of a society – a view elucidated by Epstein and Hundert [1,2] and referenced in the Introduction of this report.

Contrary to the assessment system that simply rates clinicians’ competency level at particular time points, the gap analysis incorporated into our system compares existing with expected levels of competencies empowers the receivers of medical education by acknowledging the value of their feedback. Insights culled from this group of stakeholders can inform policy-makers and administers of medical education as these decision-makers endeavor to instigate on-target improvements to bridge the pedagogical gaps. On the other hand, gap analysis facilitates the establishment of personal benchmark for clinicians, which allows them to take stock of the progress which they’ve made and titrate their goals and expectations as they continue evolving professionally. [23–25]

Additionally, the competency-based KSA scale proposed in our study can serve as a reference to guide the reform of medical licensing examinations. In the past, these examinations focused on knowledge. Today, more emphasis is placed on physicians’ professionalism and clinical skills. Pursuant to additional investigations of feasibility, the 36 indicators contained in the scale can be developed into an expanded set of criteria to assist the redesign of medical licensing examinations.

## Limitations of the study

As constrained by time and resources, the assessment rated by the 3 cohorts of graduates from the SUMC – new graduates, resident physicians, and senior physicians – was used as a proxy to gauge clinician’s professional development over time. Hence, the development trends found in this research may diverge from those in a longitudinal study that monitors the competency evolution of one single group of graduates. Secondly, the analysis of competency gaps in the study was based on participants’ self-assessment that might not corroborate fully with the assessment based on more objective measures or furnished by other key stakeholders such as patients, supervising physicians, peers, and nurses. Interpretations and extrapolations of the study findings thus need to be pursued with caution. Last but not least, the KSA-based assessment system proposed in our study was tested only among the graduates from one medical university, and needs to be further validated at additional medical institutes and in different parts of the country.

## Conclusion

A competency-based assessment system is proposed to evaluate clinician’s competency development in 3 domains: knowledge (K), skills (S), and attitude (A). The system consists of 36 competency indicators, a rating scale of 5 proficiency levels, and a gap analysis to measure competency evolution through 3 key milestones in clinician’s professional career: new graduate, resident physician, and senior physician. The competency gaps identified can provide evidence-based guide to clinicians’ own continuous development as well as future medical curriculum improvements.

## Data Availability

Data are available from the corresponding author upon reasonable request.
